# TREM2 Agonism with a Monoclonal Antibody Attenuates Tau Pathology and Neurodegeneration

**DOI:** 10.3390/cells12111549

**Published:** 2023-06-05

**Authors:** Michael Fassler, Clara Benaim, Jacob George

**Affiliations:** 1 Cognyxx Pharmaceuticals, Tel Aviv Israel and Kaplan Heart Center, Rehovot 6901658, Israel; 2 Kaplan Medical Center, 1 Pasternak St., Rehovot 76100, Israel

**Keywords:** TREM2, tauopathy, neurodegeneration, neuroinflammation, Alzheimer’s

## Abstract

TREM2 is a membrane receptor expressed on microglia that plays a pivotal role in the organization and function of these innate immune cell components within the neurodegenerated brain. Whereas TREM2 deletion has been studied extensively in experimental beta-amyloid and Tau-based models of Alzheimer’s disease, its engagement, and subsequent agonism have not been tested in the context of Tau pathology. Herein, we explored the effects of Ab-T1, an agonistic TREM2 monoclonal antibody on Tau uptake, phosphorylation, seeding, and spreading as well as its therapeutic efficacy in a Tauopathy model. Ab-T1 enhanced the uptake of misfolded Tau to microglia and induced a non-cell autonomous attenuation of spontaneous Tau seeding and phosphorylation in primary neurons from human Tau transgenic mice. Ex vivo, incubation with Ab-T1 led to a significant reduction in the seeding of Tau pathology in the hTau murine organoid brain system. Systemic administration of Ab-T1 resulted in reduced Tau pathology and propagation when hTau was stereotactically injected into the hemispheres of hTau mice. Intraperitoneal treatment with Ab-T1 lead to attenuation of cognitive decline in the hTau mice that was associated with reduced neurodegeneration and synaptic preservation with amelioration of the global neuroinflammatory program. Collectively, these observations show that TREM2 engagement with an agonistic antibody result in reduced Tau burden concomitant with attenuated neurodegeneration ascribed to the education of resident microglia. These results may suggest that despite the opposing results with regard to the effect of TREM2 knockout in experimental Tau-based model systems, receptor engagement and activation by Ab-T1 appears to possess beneficial effects with respect to the various mechanisms mediating Tau-driven neurodegeneration.

## 1. Introduction

The pathological hallmarks of the Alzheimer’s disease (AD) brain encompass extracellular senile plaques comprising amyloid-β peptides as well as intracellular inclusions of misfolded Tau protein [1,2]. Tau pathology is evident in Aβ plaque-associated neuronal processes as neuritic plaque tau, but also as neurofibrillary tangles and as neuropil threads. Tau inclusions are associated with neurodegeneration in AD, with supportive evidence arising from immunohistochemical studies showing an association between the extent of tau pathology and cognitive impairment [3,4], which is not evident with respect to Aβ plaque burden. These pathological findings are corroborated by PET imaging studies that associate Tau pathology with cognitive deficits [5], as well as with brain atrophy [6]. CSF-derived biomarkers reflective of misfolded/phosphorylated Tau burden are also associated with the clinically meaningful role of Tau misfolding in the pathogenesis of neurodegeneration [7].

TREM2 is a membranal receptor expressed in microglia exclusively in the CNS, whereas it is also found peripherally on dendritic cells and macrophages as well as in osteoclasts [8,9]. The strongest data supporting the role of TREM2 in neurodegenerative diseases arise from genome-wide association genetic studies that point to an increased risk of AD conferred by impaired function variants of this receptor [10]. The robust presence of TREM2 on microglia, the major innate immune effector in CNS, thus further supports the role of neuroinflammation in neurodegeneration.

Inconclusive data are available from multiple TREM2 transgenic murine models with regard to the effect on AD pathology. However, several acceptable themes do seem to prevail and relate to the requisite role of membrane-bound TREM2 in encircling the amyloid plaques thereby altering their morphology to a more compact architecture and an attenuated neuritic pathology [8].

The data with regard to the role of TREM2 in the development of Tau pathology appears complex with data coming principally from models harboring a deletion of the gene encoding the receptor. Bemiller et al. [11] previously described a tauopathy model where TREM2 ablation accelerated Tau phosphorylation and aggregation at the early stage without data on later stages. In the P19 model, however, the genetic absence of TREM2 was associated with a late attenuation of brain atrophy and neuronal loss without influencing Tau phosphorylation or aggregation [12]. In a later study using the same murine model, heterozygous expression but not full deletion of TREM2 was associated with an accelerated Tau pathology [13]. Two recent papers [12,14] studied the role of TREM2 deletion in mixed amyloid and Tau pathologies. In the first, Leyns et al. [12] used an AD model where human-derived Tau was injected into mice with preexistent amyloid pathology. In this model, TREM2 ablation was associated with an early increase in p-Tau surrounding amyloid plaques, with no data on later stages. Recently, to gain insight into the role of TREM2 in the different stages of Tau-derived amyloid pathology, Lee et al. [14] crossed TREM2 KO with the P301L mice harboring the Tau mutation. This double transgenic model was tested with and without the introduction of the PS2APP transgene to test the impact of TREM2 deletion in the presence or absence of amyloid pathology. Collectively, their data support the role of TREM2 in protecting against tau spreading and accumulation and the consequent neuronal damage in the presence but not in the absence of beta-amyloid pathology.

The vast majority of experimental data relating to the effects of TREM2 on Tau-mediated neuroinflammation and neurodegeneration comes from gene ablation or impaired function transgenic mouse models. However, agonism/activation of membrane-bound TREM2 is not necessarily expected to yield opposing effects on amyloid and Tau pathologies. Others and our group have recently described the use of monoclonal antibodies targeting various domains in the extracellular domain of TREM2 as means of activating microglia [15,16,17,18]. In general, the antibodies have demonstrated protective effects on various cellular features of neurodegeneration in 5xFAD and APPS1 mice [8]. However, the role of TREM2 engagement and activation in affecting Tau pathology has not yet been tested. Herein, we used our previously reported TREM2 agonistic monoclonal antibody, Ab-T1 in a collective set of in vitro, ex vivo, and in vivo studies. We show that microglial TREM2 engagement with Ab-T1 is associated with reduced Tau phosphorylation, spreading, and aggregation with consequent beneficial effects on neurodegeneration.

### 1.1. Materials and Methods

#### Subjects and Tissue Sampling

This study was performed at Kaplan Medical Centre in Rehovot, Israel under appropriate Institutional Review Board approval. Blood samples were obtained from the Israel National Blood Services (MDA) except for CSF and brain specimens included in the study which were collected at the Cambridge Brain Bank supported by the NIHR Cambridge Biomedical Research Centre.

## 2. Animals

hTau.K257T/P301S animals kindly provided by Dr. Rosenmann (Hebrew University) were bred in-house (mutations were analyzed using specific primers for PCR genotyping) [19]. All housing, breeding, and procedures have been reviewed and approved by “The Israel Board for Animal Experiments” and are in compliance with “The Israel Animal Welfare Act”. Animals were housed under standard laboratory conditions, air-conditioned and filtered (HEPA F6/6) with adequate fresh air supply (minimum 15 air changes/hour). Animals were kept in a climate-controlled environment. Temperature range was 20–240 °C and relative humidity range was 30–70% with a 12-h light and 12-h dark cycle.

### 2.1. Immunohistochemistry

Brain tissue sections from hTau.K257T/P301S mice were post-fixed in 4% formalin for 48 h, dehydrated in ethanol, and embedded in paraffin. A series of sections were stained for either anti-Iba-1 (Abcam, ab178847, Cambridge, UK), anti-pTau antibody (Ser404) (Abcam, ab109390), anti-caspase 3 (Abcam, ab184787, Cambridge, UK) or mouse IgG (Abcam, ab37355, Cambridge, UK).

Immunohistochemical staining was performed on 4 µm sections using the Leica Bond III system (Leica Biosystems Newcastle Ltd., Newcastle, UK). Tissues were pretreated with epitope retrieval solutions (ER, Leica Biosystems Newcastle Ltd., UK) followed by 30 min incubation with primary antibodies anti-Iba-1 (1:1500), anti-pTau (1:200), anti-caspase 3 (1:1000) or mouse IgG (1 μg/mL). The Leica Refine-HRP kit (Leica Biosystems Newcastle Ltd., UK) was used for detection followed by counter-stain with hematoxylin or 1% cresyl violet.

### 2.2. Image Analyses

Photographs were taken with VENTANA DP 200 slide scanner auto cell imaging system (Roche, Switzerland) using Ventana image viewer software. Regions of interest in each slide were outlined by an investigator blinded to the animal clinical data. A counting frame of 80 × 80 μm at 20× magnification was randomly selected in each region (20 frames in each region) for counting neuronal cells by separate tags for different morphological stages (live vs. damaged neurons), caspase 3 positive cells, neurofibrillary tangles, or microglia by separate tags for different morphologically microglia stages (activated vs. nonactivated). To obtain composite average of cells in brain slices, data were averaged from four to six adjacent blocks of tissue.

### 2.3. Lysate Preparation

Brain and cell lysates were prepared in hypotonic buffer (0.01 M Tris, pH 7, 1 mM EDTA, 1 mM EGTA) freshly supplemented in protease inhibitor cocktail (P8340, Sigma)), incubated on ice for 30 min, snap frozen, thawed, followed by pellet resuspension in STE lysis buffer (150 mM NaCl, 50 mM Tis-HCl, pH 7.6, 2 mM EDTA, 1% Triton-X 100) and incubated on ice for 20 min before clarifying and supplemented with Laemmli sample buffer for SDS-PAGE protein separation.

### 2.4. Tau Brain Extraction

Hippocampi and cortexes from human brain tissue were homogenized in buffer 1 (high salt/sucrose buffer 10 mM Tris-HCl, pH 7.4, 800 mM NaCl, 10% sucrose (*w*/*v*), 1 mM EGTA) supplemented with phosphatase inhibitor cocktail sets I and II (Calbiochem, San diego, CA, USA), complete protease inhibitor cocktail EDTA-free (Roche, Indianapolis, IN, USA) and 1 mM PMSF (Sigma, St. Louis, MO, USA) and extracted in cold buffer (10X *w*/*v*) using a Potter-Elvehjem homogenizer.

The extracts were then centrifuged at 20,000× *g* for 30 min at 4 °C and then supernatant was divided to generate the heat-stable soluble fraction and the sarkosyl insoluble fraction. An aliquot of the soluble, supernatant fraction (300–400 µL) was stored frozen at −80 °C while the slow-speed pellet is discarded. The remaining 200 µL of the low-speed supernatant is treated with Sarkosyl (N-lauroylsarcosine, Sigma L-7414), adjusted to 1% sarkosyl (*v*/*v*), and incubated for 60 min at 4 °C with rocking. This extract is then centrifuged at 100,000× *g* (31,400 rpm) for 1 h at 4 °C and the resulting sarkosyl-insoluble pellet is rinsed using high salt/sucrose buffer (buffer1) and re-centrifuged. Pellet is then resuspended in 200 µL of buffer 2 (50 mM Tris-HCl, pH 7.4 buffer containing 2.3% SDS, 1 mM EGTA, 1 mM EDTA), supplemented with phosphatase inhibitor cocktail sets I and II (Calbiochem, San Diego, CA, USA), complete with protease inhibitor cocktail EDTA-free (Roche, Indianapolis, IN, USA) and 1 mM PMSF (Sigma, St. Louis, MO, USA). The pellet is sonicated in water bath for 30 min at RT and then aliquots are stored frozen at −80 °C. This comprised the sarkosyl-insoluble fraction.

### 2.5. Immunofluorescence Staining in Cell Culture

Primary culture of neurons from day P0 newborn mice (cortex) were prepared as described previously [20]. Primary neurons of hTau.K257T/P301S mice were seeded on coverslips coated with poly-L-lysine (Sigma). One week later, BV-2 microglia cells (ICLC ATL03001, ICLC cell bank) treated with Ab-T1 (10 µg/mL), control IgG4 (10 µg/mL) or PBS with pathological pTau mix from human AD brains or PBS (O.N incubation for activation) were added to the wells, creating co-cultures of neuronal cells and microglia (3 wells for each sample). Seven days later, cells were washed with PBS and fixed using 4% paraformaldehyde. Fluorescent quenching was performed using 0.4 mg/mL of 10 mM NaBH4. Cells were permeabilized using 1% Triton X-100 and blocked in PBS supplemented with 10% goat serum and 0.1% triton X-100 for 1 h at room temperature. Cells were incubated with mouse anti-Tau (1:1000) and rabbit anti-synaptophysin (1:300) as primary antibodies (diluted in PBS supplemented with 1% goat serum as blocking buffer) overnight at 4 °C followed by adding Alexa fluor 647 anti-rabbit IgG (1:500) or Alexa fluor 488 anti-mouse IgG (1:300) secondary antibody in blocking buffer for additional 1–2 h at room temperature. Cells were rinsed twice with ice-cold PBS and fixed in 4% v/v paraformaldehyde for 12 min at room temperature. Cells on coverslips were washed 3 times in ice-cold PBS and laid down on a slide with a drop of mounting buffer (DAPI Fluoromount-G, SouthernBiotech) before being visualized on a confocal laser scanning microscope (TCS SP8, Leica).

### 2.6. Immunofluorescent Staining of Brain Tissues

Slices were rinsed in 0.3% Triton X-100/PBS (3 × 5 min). In order to prevent non-specific binding, slices were pre-incubated with blocking buffer (1% bovine serum albumin (BSA), 0.2% Triton X-100 and 5% goat serum in PBS) for 1.5 h at room temperature. Slices were incubated with NeuN antibody (Sigma, MAB377; 1:200) and synaptophysin antibody (Abcam ab32594; 1:300) overnight at 4 °C in incubation buffer (0.1% BSA and 0.01% Trition X-100). Slides were rinsed with 0.3% Triton X-100 in PBS (3 × 5 min) followed by incubation with Alexa Fluor 488 anti-mouse IgG (Abcam, ab150113; 1:300) and Cy5 anti-rabbit IgG (Abcam, ab6564; 1:500) in blocking buffer for additional 2 h at room temperature. Slides were washed 3 times in PBS and a drop of mounting buffer (DAPI Fluoromount-G, SouthernBiotech) was added before being visualized on a confocal laser scanning microscope (TCS SP8, Leica).

### 2.7. Western Blotting for TREM2 Detection

An amount of 30–25 μg of cell lysate/CSF was loaded in each well for SDS-PAGE protein separation (reduced conditions) on Nuphage 4–12% gels (ThermoFisher Scientific, Cat No. NP0322BOX, Carlsbad, CA, USA). Samples were transferred to 0.2 μm nitrocellulose membranes (WhatmanProtan BA83, Cat No. 10401380, Marlborough, MA, USA) and incubated at 4 °C for 12 h with mouse Ab-T1 (5 μg/mL) or mouse IgG (5 μg/mL control) as primary antibodies diluted in 1% BSA/TBST followed by goat anti-mouse conjugated to HRP (Jackson, Cambridge, UK, 1:10,000) for additional 1 h incubation at room temperature. Membranes were developed using Fusion Solo 7S imager system (Vilber, Collegien, France).

### 2.8. Endogenous Phosphorylated Aggregated Tau Levels in hTau.K257T/P301S Brains Ex Vivo

Oxygen was added to slicing medium (MEM supplemented with 10 g/L D-glucose and 1 mM HEPES (pH 7.2)) with 95% O_2_/5% CO_2_ and incubated on ice before use. Ten % FBS plating medium (neurobasal supplemented with 10 mM HEPES, 1 × B-27, 400 μM L-glutamine, 600 μM GlutaMAX, 60 U/mL penicillin, 60 μg/mL streptomycin, 10% FBS) was added into each well of a 6-well plate and placed in an incubator (36.5 °C, 5% CO_2_) for at least 1 h prior to explantation to allow the medium to warm and pH to adjust. Mouse brains were harvested and placed in a Petri dish to make a single coronal cut to separate cerebrum from cerebellum. Specimens were glued to the vibratome stage and placed inside the vibratome bath, followed by addition of ice-cold oxygenated slicing medium into the vibratome bath until the point where the olfactory bulbs/frontal lobe are just submerged. Specimens were sliced to 400 μM thick slices containing hippocampus/thalamus and each nascent slice was collected into 6-well plates containing ice-cold, oxygenated slicing medium. Millipore mash inserts were placed into each of the 6-well plate wells with sterile forceps and slices were transferred onto them. Slicing medium that was transferred along with slices was removed using a Pasteur pipette and plates were placed in a humidified incubator set at 5% CO_2_ and 37 °C. Immediately after plating or slice recovery 15 μg/mL pathological tau mix from human AD brains was added with the following treatment of Ab-T1, control IgG, or PBS (10 μg/mL) to each slice. PBS alone was added as sham inoculation instead of pathological tau as control. Next day, medium was replaced with maintenance medium (5% FBS plating medium) and slices were incubated for 10 additional days before protein extraction. Different sections treated with Ab-T1 and control IgG were homogenized 10 days later using a tissue homogenizer (TH tissue homogenizer, Omni) with 10-fold volume of 50 mM Tris-HCl homogenization buffer, pH 7.4 freshly supplemented in protease inhibitor cocktail and phosphatase inhibitors and incubated on ice for 20 min. Homogenized tissue was centrifuged 20,000× *g* for 20 min at 4 °C and supernatant was collected as crude tau fraction (contains soluble and insoluble Tau). Thirty micrograms of lysates per sample were loaded in each well for SDS-PAGE protein separation on Nuphage 4–12% gels (ThermoFisher Scientific, Cat No. NP0322BOX). Samples were transferred to 0.2 μm nitrocellulose membranes (WhatmanProtan BA83, Cat No. 10401380) and incubated at 4 °C for 12 h with rabbit anti-phospho (serine 404) tau (Abcam, Cat #: Ab92676; 1:500), mouse anti-mouse specific tau (clone T49) (Millipore, Cat #: MABN827; 1:2000) or 1 h with mouse anti-tubulin (BioLegend, Cat #: 8012021, San Diego, CA, USA; 1:1000) as primary antibodies diluted in 1% skim milk/TBST followed by goat anti-mouse conjugated to HRP (Jackson, 1:10,000) for additional 1 h incubation at room temperature. Membranes were developed using Fusion Solo 7S imager system (Vilber, France) and the volume intensity (pixels) normalized to Tubulin for tau phosphorylation and endogenous tau spreading quantification was calculated using Fusion Solo S software (Vilber, France).

### 2.9. The Effect of Ab-T1 on Tau Spreading in the hTau Murine Model

To evaluate if Ab-T1 treatment inhibits pTau spreading, hTau transgenic mice were stereotactically injected with pathological tau from human brains with Alzheimer’s disease, 9 females (3 groups of *n* = 3). H-Tau.K257T/P301S mice (9 weeks of age) were anesthetized by Isoflurane, and stereotactically injected with human pathological Tau mix from human AD brains (hippocampus 2.5 μg + overlaying cortex 1 μg) in PBS (total of 3.5 μg of pTau per brain). Control sham hTau.K257T/P301S animals received sterile PBS (*n* = 2). A single needle insertion (coordinates: −2.5 mm relative to bregma, 2.0 mm from midline) into the right hemisphere was used to target the inoculum to the hippocampus located at a depth of 2 mm below the dura and an additional single needle insertion into overlaying cortex (coordinates: −1.4 mm from the skull). Material was injected via a 10 μL Hamilton syringe at a rate of 0.4 μL per min (3.5 μL total volume) with the needle in place (33G) for ≥10 min at each target. Animals were inoculated at the right hemisphere unless otherwise indicated. Mice were treated i.p with 10 mg/kg of mAb-T1, control mouse IgG or PBS as sham control once a week for 2 months. Animals were sacrificed at 8 weeks from beginning of experiment by overdose with ketamine/xylazine followed by transcardial perfusion with PBS. For histological studies, the brain was removed and underwent overnight postfixation with neutral buffered formalin (Thermo Fisher Scientific, Waltham, MA, USA), before being processed and embedded.

## 3. Treatment with Ab-T1 in the hTau.K257T/P301S AD Animal Model

A total of 26 males (3 groups of *n* = 10, 9, 9) hTau.K257T/P301S mice (10–11 months of age) were anesthetized by Isoflurane, intraperitoneal (i.p.) injected with anti-CD4 (clone GK1.5, InVivoMAb, BE0003-1) for CD4 cell depletion 6 days pre-treatment. Mice were treated i.p with humanized Ab-T1 (1 mg/kg and 10 mg/kg, *n* = 9, 9) and human IgG (10 mg/kg, *n* = 10) once every two weeks for 3 months. A behavior test (MWM) was conducted before they were sacrificed at 12 weeks post injections by overdose with ketamine/xylazine. For histological studies, the brain was removed, and the left hemisphere underwent overnight post-fixation with neutral buffered formalin (Thermo Fisher Scientific), before being processed and embedded in O.C.T compound (Ref 4583, Tissue-Tek, Torrance, CA, USA) for cryosectioning and immunostaining (Cresyl violet staining (CEA500, ScyTek Laboratories, Logan, UT, USA) and immunohistochemistry. For biochemical studies, right hemisphere brain tissues were immediately frozen after removal and stored at −80 °C until used. CSF and blood were taken from all 25 animals. Blood was examined for hematological and biochemical parameters such as cytokines (RayBio^®^ Mouse Cytokine Antibody Array Kit, Peachtree Corners, GA, USA), human IgG4 (ELISA assay kit, Cat #: BMS2095 Invitrogen, Carlsbad, CA, USA), and soluble TREM2 levels (ELISA assay kit, Cat #: #LS-F7884, LifeSpan BioSciences Inc., Seattle, WA, USA).

### 3.1. Proteomic Profile of Cytokines in Brains of hTau.K257T/P301S Mice Treated with Ab-T1

Protein levels in brain lysates were detected using a protein array glass chip with a fluorescence detection assay kit (RayBio^®^) following manufacturer protocol. Brain lysate pool of 5 hTau.K257T/P301S mice from each group of treatment (10 mg/kg of Ab-T1 or control IgG) was added to the protein microarray glass chip prepared following manufacturer protocol. Microarray glass chip was scanned on a microarray fluorescence scanner for proteomic profile of cytokines (FAS ligand, Fractalkine, IL1-alpha, IL1-beta, IL4, IL5, IL6, IL10, IL12-p40/p70, IL17, KC, Leptin R, Leptin, MCP-1, SDF-1 alpha TARC TPO, and VCAM-1 protein levels detected by RayBio^®^ Mouse Cytokine Antibody Array Kit, Peachtree Corners, GA, USA)

### 3.2. Assessment of Cognitive Function by the Morris Water Maze (MWM)

The Morris water maze task was performed as described previously [18].

## 4. Statistical Analysis

Values shown in the figures are presented as mean +/− SEM unless otherwise mentioned. P values for determination of the statistical significance of differences were calculated by means of paired, two-tailed Student’s *t* test, and two-tailed Mann–Whitney U test. Statistical analysis was performed using Prism 8. *p* = 0.05 was defined as the level of significance unless otherwise indicated in vitro data shown represent three independent experimental repeats with triplicate technical repetition.

## 5. Results

Ab-T1 binds specifically TREM2 and recognizes two consistent bands representing mature and immature forms in the brain and CSF of AD patients.

**Figure 1:**
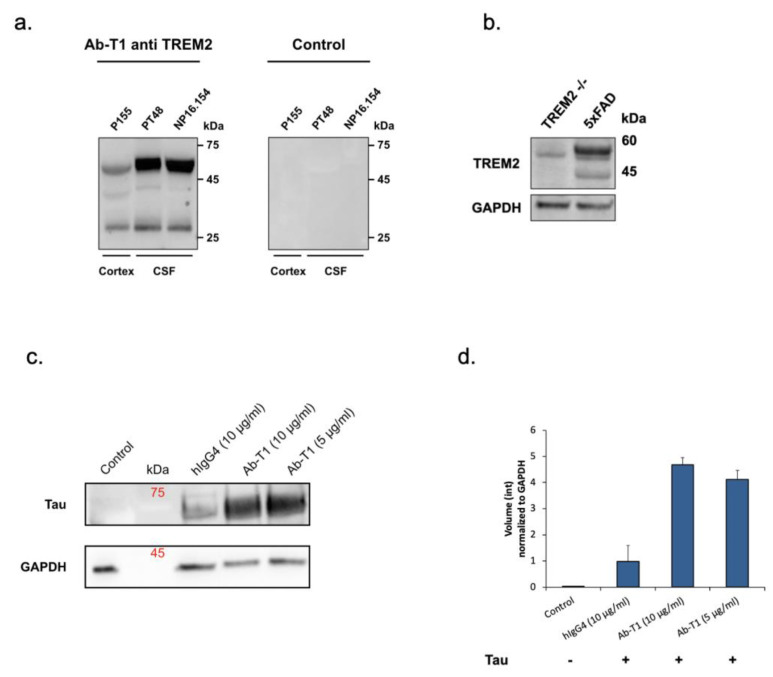
Binding characteristics of Ab-T1 and pTau uptake assays. (**a**) Western blots of soluble hTREM2 detection in human CSF and entorhinal cortex from Alzheimer patients (internal patient ID: P155, PT48, and NP16.154) using murine Ab-T1 (left panel) and mouse IgG (right panel) as control IgG antibody. (**b**) Ab-T1 binding to 5xFAD and TREM^-/-^ 5xFAD (KO) mice brain extracts. (**c**,**d**) Uptake of aggregated p-Tau by microglia cells (BV-2) as measured by Western blot. (**d**) Volume intensity (pixels) normalized to GAPDH from Western blot images analyzed using Fusion Solo S software (Vilber, France).

Ab-T1 was shown to bind its target TREM2 in the entorhinal cortex of human brains (cortex) and CSF from Alzheimer’s disease patients seen in Western blot (Figure 1a—left panel). There was no binding to TREM2 in a negative control IgG staining (Figure 1a—right panel). To further confirm the specificity, Ab-T1 was shown to bind its target TREM2 in 5xFAD mice but not in TREM^-/-^ 5xFAD (KO) mice brain extracts seen in the Western blot assay (Figure 1b). It can be clearly observed that several bands representing several TREM2 fragments are detected by the antibody representing soluble clipped forms in the extracellular space and internalized degraded form of the membranous protein.

### 5.1. Ab-T1 Promotes Microglial Uptake of pTau

Western blot assay was used to detect and measure microglia uptake of aggregated phosphorylated Tau (p-Tau) (Figure 1c,d). BV-2 microglia cells treated with Ab-T1 had an increase in cellular uptake of p-Tau (Ab-T1; 5 μg/mL: volume intensity 4.12 and 10 μg/mL: volume intensity 4.67) compared to cells with IgG control treatment (hIgG4; volume intensity 0.97) seen in Figure 1d.

### 5.2. Inhibition of Tau Spreading/Aggregation in Primary hTau Transgenic Neurons by Microglia Primed with Ab-T1

**Figure 2:**
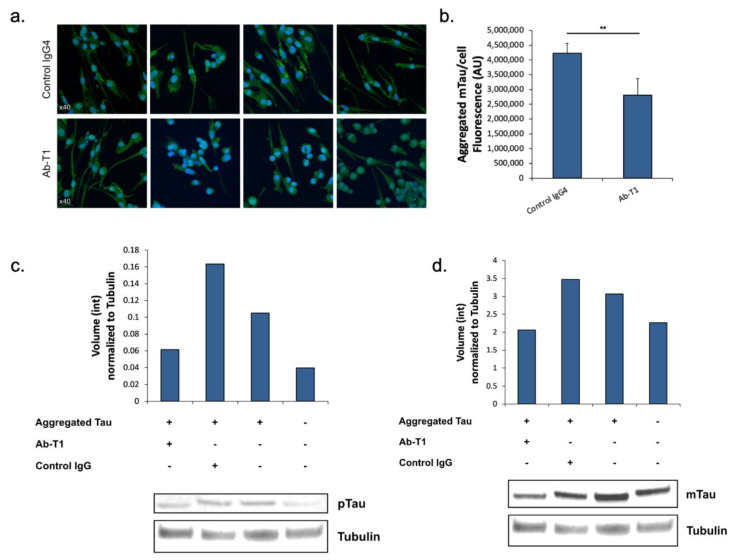
Attenuated pTau pathology in a coculture experiment in vitro and in an ex vivo organoid brain slice system. (**a**) Representative micrographs of cocultured mutant (K257T/P301S) primary neurons and Ab-T1 or control IgG4 treated microglia processed for immunofluorescence using the anti-Tau (T49 clone), which detects misfolded murine pTau. (**b**) Quantification of the fluorescence intensity of endogenous misfolded mouse Tau (anti-mouse Tau staining) per single neuron in mutant primary neurons cocultured with Ab-T1 or control IgG4 treated microglia. AU—arbitrary units measuring fluorescence intensity (**b**) Two-tailed Student’s *t* test, ** *p* < 0.05). Magnification ×40. (**c**) Total phosphorylated tau protein levels in ex vivo brain sections treated with Ab-T1 or control IgG (10 µg/mL). (**d**) Total endogenous tau protein levels in ex vivo brain sections treated with Ab-T1 or control IgG (10 µg/mL). Mean endogenous tau protein levels (mTau) and mean phosphorylated tau protein levels in each treatment group. Y-axes represent volume intensity (pixels) levels of total tau or phosphorylated tau normalized to tubulin from Western blot images analyzed using Fusion Solo S software (Vilber, France).

Here, we tested the hypothesis that microglia treated with Ab-T1 have a protective role in neurons via activation of these microglia, influencing the accumulation of misfolded aggregated pTau within neurons of hTau.K257T/P301S mice. Activated microglia with Ab-T1 have the ability to reduce significantly the accumulation of misfolded pTau in mutant primary neurons from hTau.K257T/P301S mice compared to control IgG4 treatment in seeded primary neurons (Figure 2a). There were significantly lower levels of murine endogenous aggregated pTau stained with the specific anti-mouse Tau antibody (not human) seen in neurons co-cultured with Ab-T1-treated murine microglia (Figure 2b; Ab-T1) compared to the control IgG-treated group (Figure 2b; control IgG4) as seen and fluorescently measured.

### 5.3. Inhibition of Tau Spreading/Aggregation in Ex Vivo Brain Slices from hTau Transgenic Mice Treated with Ab-T1

Here, we analyzed and quantified p-Tau protein and total murine endogenous tau protein levels compared to tubulin protein levels in ex vivo brain slices from hTau.K257T/P301S mice treated with Ab-T1 or control IgG. Protein levels of phosphorylated tau or total endogenous tau (mouse tau) normalized to total tubulin levels in hTau.K257T/P301S transgenic mice (AD/Tauopathy) treated with Ab-T1 or hIgG4 were detected and analyzed using Western blot immunoassay (Figure 2c). Brain sections treated with Ab-T1 showed lower levels of tau phosphorylation (Figure 2c—volume intensity = 0.06) compared to control IgG treatment (Figure 2c—volume intensity = 0.16). Furthermore, brain sections treated with Ab-T1 showed lower levels of mouse endogenous tau protein (Figure 2d—volume intensity = 2.06) compared to control IgG treatment (Figure 2d—volume intensity = 3.46).

### 5.4. In Vivo Treatment with Ab-T1 Ameliorates Spreading and Seeding of pTau

The objective of the study was to evaluate if intraperitoneal Ab-T1 treatment (once/week) inhibits pTau spreading in AD/tauopathy hTau.K257T/P301S mice seeded with pathological Tau from human brains of patients with Alzheimer’s disease (AD-Tau).

**Figure 3:**
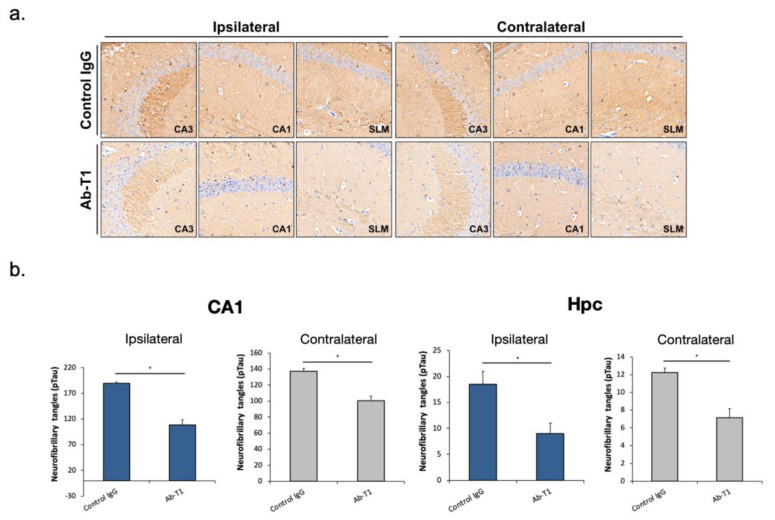
Ab-T1 inhibits seeding and spreading of Tau pathology in vivo. (**a**) High magnification of hyperphosphorylated, anti-pTau (ser404) antibody-positive tau pathology (neurofibrillary tangles) in the ipsilateral and contralateral CA1, CA3, and striatum lacunosum-moleculare (SLM) region of the hippocampus demonstrating neuritic plaque tau pathology; ×20 (**b**) quantification of neurofibrillary tangles—tau pathology positive staining cells reveals treatment with Ab-T1 significantly reduced the amount of anti-pTau (ser404)-positive tau pathology on the ipsilateral side, and same trend of reduction of tau pathology is shown on the contralateral side. Ab-T1 treatment group was compared to the corresponding IgG isotype control group (Mann–Whitney U test; * *p* < 0.05). In order to obtain composite average of tau pathology in brain slices, cells from six adjacent blocks of tissue were quantified.

IHC staining of hyperphosphorylated Tau with the anti-pTau (ser404; ab109390, Abcam) antibody reveals abundant tau pathology in AD-tau injected hTau.K257T/P301S mice 2 months post-injection. Seven weeks post-treatment, mice treated with 10 mg/kg of Ab-T1 showed lower levels of neurofibrillary tangles (pTau) in both ipsilateral and contralateral brain sections (Figure 3a—lower panels) compared to control IgG treatment (Figure 3a—upper panels). There was a significantly smaller number of neurofibrillary tangles (pTau) in Ab-T1-treated mice (Figure 3b Hpc; Ipsilateral: 108.3 ± 9.83, Contralateral: 100.57.3 ± 5.78) compared to control IgG-treated group (Figure 3b Hpc; Ipsilateral: 189.5 ± 2.5, Contralateral: 137.33.3 ± 3.38) at the hippocampus (Hpc) area and specifically at the CA1 region (Figure 3b CA1; Ipsilateral: 9 ± 2; Contralateral: 7.14 ± 1.01 for Ab-T1-treated mice and Ipsilateral: 18.5 ± 2.5; Contralateral: 12.25 ± 0.47 for control IgG-treated mice) as measured in the ipsilateral coronary brain sections (Figure 3—* *p* < 0.05).

### 5.5. Ab-T1 Improves Cognition in hTau.K257T/P301S Mice

**Figure 4:**
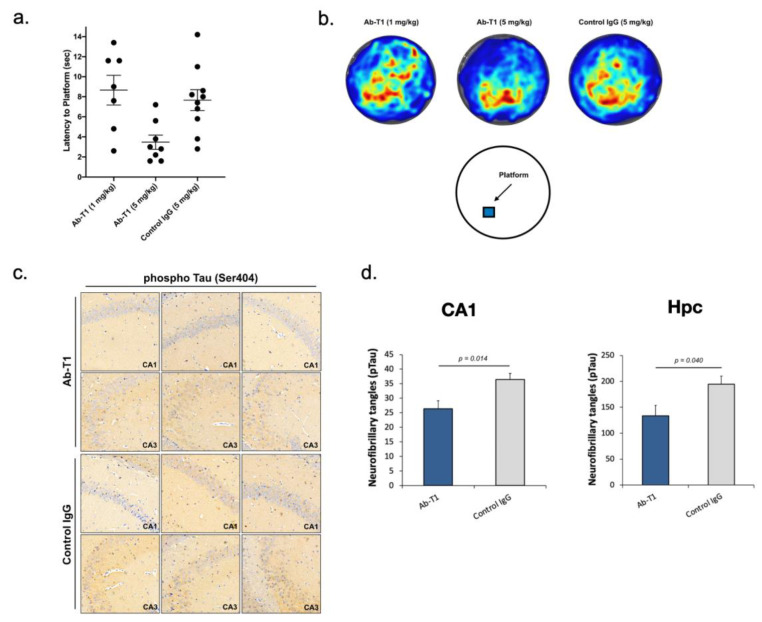
Cognitive decline assessment using Morris water maze test and reduction of neurofibrillary tangles in the brain. (**a**) Control IgG-treated hTau.K257T/P301S mice compared to Ab-T1-treated mice showed cognitive impairment as indicated by the increase in latency to platform in the MWM probe test. (**b**) Heat map average group images of time mice spent in different areas of the MWM pool. Error bars represent standard error of the mean. (**c**) Immunohistochemical images of neurofibrillary tangles in hippocampal area (CA1 and CA3). (**d**) Total number of neurofibrillary tangles in both control IgG and Ab-T1 treatment groups in hippocampus and CA1 (Hpc, two-tailed Student’s t test, *p* = 0.040), (CA1, two-tailed Student’s *t* test, *p* = 0.014). Magnification ×20.

hTau.K257T/P301S mice at 10–11 months of age, a time point where these mice are known to exhibit neurofibrillary tangles (NFT) pathology and memory deficits within the brain were intraperitoneally injected with different doses of Ab-T1 (1 and 5 mg/kg), a human IgG antibody (5 mg/kg) and or IgG control twice a month (*n* = 9–10 per group). The dose was selected based on our previously published data as well as using pharmacokinetic and pharmacodynamics studies showing this dose completely saturates levels of murine soluble TREM2 in the serum. Morris water maze probe test behavioral tests were conducted to examine their cognitive decline (Figure 4a,b). Twelve weeks post-treatment, mice treated with 5 mg/kg of Ab-T1 showed a significantly shorter latency to escape onto the hidden platform in the Morris water maze probe test (Figure 4a, 3.48 ± 0.70 s) as compared to 5 mg/kg of human control IgG (Figure 4a, 7.66 ± 1.04 s, *p* = 0.006) treated control mice. There was no significant difference between human IgG control mice and 1 mg/kg Ab-T1-treated mice. Heat map images of the average time mice spent in different areas of the pool in the MWM probe test showed that Ab-T1-treated mice spent the majority of time in the southwest area (Figure 4b, platform area—middle image) while human IgG-treated mice did not have a preferable area (Figure 4b, right image).

### 5.6. Ab-T1 Attenuates Phospho-Tau in the Hippocampus of hTau.K257T/P301S Mice

Here, we analyzed and quantified neurofibrillary tangles (pTau staining) in the hippocampal region in a number of consecutive sagittal brain slices of treated (5 mg/kg Ab-T1 or control IgG) hTau.K257T/P301S animals.

Immunostaining images of neurofibrillary tangles in the hippocampal region area show a lower number of neurofibrillary tangles in Ab-T1-treated animals compared to IgG-treated animals (Figure 4c,d). Neurofibrillary tangles were quantified using magnified images (10 frames in each region per slice) (Figure 4c). There was a significantly lower number of neurofibrillary tangles in Ab-T1-treated animals as compared to IgG-treated animals in both hippocampal regions and specifically at the CA1 (Figure 4c,d).

Ab-T1 ameliorates neurodegeneration/neuronal loss and preserves synaptic markers in a dose-dependent manner in the hippocampus of hTau.K257T/P301S mice.

**Figure 5:**
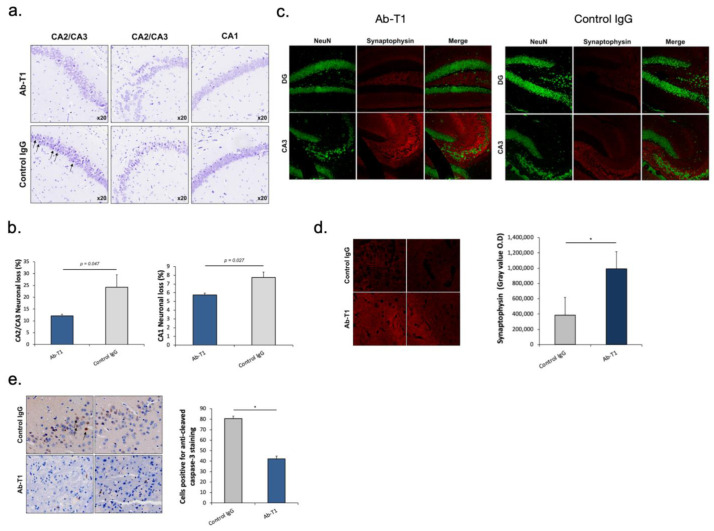
Ab-T1 reduces neuronal loss in hippocampus of tauopathy Alzheimer mice. (**a**) Immunohistochemical images of Nissl staining in the brain of hTau.K257T/P301S mice. (**b**) Neuronal loss (dark Nissl staining) in CA1 and CA2/CA3 hippocampal area in both control IgG and Ab-T1 treatment groups (CA1, two-tailed Student’s *t* test, *p* = 0.027; CA2/CA3, two-tailed Student’s *t* test, *p* = 0.047). Magnification ×20. (**c**) Immunofluorescent staining was performed to detect the markers of neuronal cells (NeuN) and pre-synaptic terminals (Synaptophysin) of hTau.K257T/P301S mice. (**d**) Optical density of synaptophysin area in hTau.K257T/P301S-treated mice. * *p* < 0.01 indicates significant differences between the groups. (**e**) Immunohistochemical images of caspase 3 in the brain of hTau.K257T/P301S mice and total number of cells positive for anti-cleaved caspase 3 staining in both control IgG and Ab-T1 treatment groups (B, two-tailed Student’s *t* test, * *p* < 0.05). Magnification ×20.

Nissl staining images of the hippocampal area show a decrease in neuronal loss in Ab-T1-treated animals compared to IgG-treated animals (Figure 5a,b). Neuronal loss was quantified using magnified images (10 frames in each region per slice) (Figure 5a). There was a significantly lower number of neuronal cell death (dark purple neuronal cells) in Ab-T1-treated animals as compared to IgG-treated animals in the hippocampal region (Figure 5a,b). Furthermore, Ab-T1-treated mice show higher levels of presynaptic terminals compared to control IgG-treated mice measured by the optical density of synaptophysin staining (Figure 5c,d). Ab-T1 significantly ameliorated the reduction of the number of NeuN (+) cells (Figure 5c, left panel) and signal intensity of synaptophysin area (Figure 5d) in hTau.K257T/P301S mice, compared with control IgG-treated hTau.K257T/P301S mice (Figure 5c, right panel). Immunostaining images of caspase 3 positive cells in the brain area show a lower number of apoptotic cells in Ab-T1-treated animals compared to IgG-treated animals (Figure 5e), quantified using magnified images (10 frames in each region per slice). There was a significantly lower number of caspase 3 positive cells in Ab-T1-treated animals as compared to IgG-treated brains of hTau.K257T/P301S animals (Figure 5e).

Ab-T1 treatment reduces the number of activated microglia and neuroinflammation in hTau.K257T/P301S mice.

**Figure 6:**
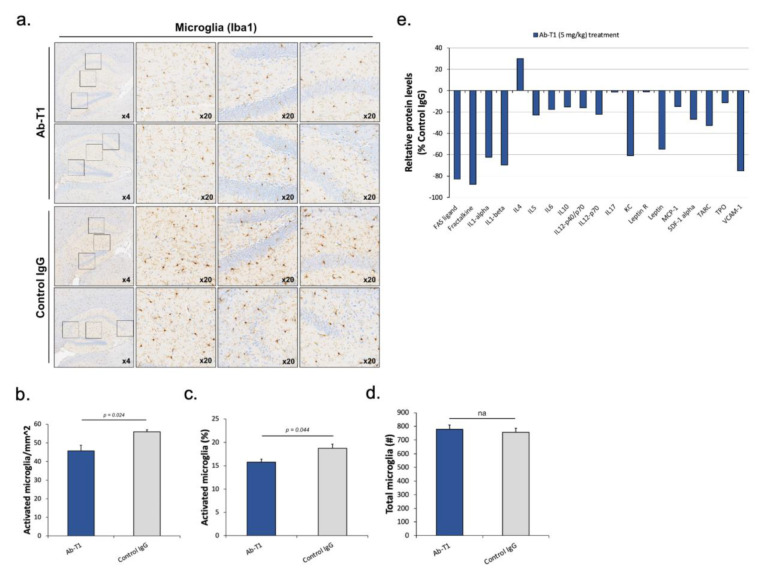
Ab-T1 reduces the number of microglia activated in the brain. (**a**) Activated microglia in hippocampal area (**b**) measured as a percent of total microglia. (**c**) Total activated microglia average in sagittal brain slice area. (**d**) Total number of microglia in both control IgG and Ab-T1 treatment ((**b**), Two-tailed Student’s *t* test, *p* = 0.044); ((**c**), two-tailed Student’s *t* test, *p* = 0.024). Magnification ×4, ×20. (**e**) FAS ligand, Fractalkine, IL1-alpha, IL1-beta, IL4, IL5, IL6, IL10, IL12-p40/p70, IL17, KC, Leptin R, Leptin, MCP-1, SDF-1 alpha TARC TPO, and VCAM-1 protein levels were detected using a mouse cytokine antibody array chip in purified brain homogenates in Ab-T1 compared to control IgG treated mice. The y-axis represents the relative % of normalized protein levels in Ab-T1-treated group relative to control IgG-treated group (read as signal intensity output from microarray fluorescence scanner). *n* = 5, 5.

We analyzed and quantified activated microglia in the total brain and hippocampal region in a number of consecutive sagittal brain slices of treated (5 mg/kg of Ab-T1 or control IgG) hTau.K257T/P301S animals. Two investigators blinded to the animal clinical data, quantified microglia activation by its morphological characteristics to estimate the total number of activated microglia in the brain and hippocampal region (Figure 6a–d). Immunostaining images of microglia in the hippocampal region and total brain area show a lower number of microglia in Ab-T1-treated animals compared to IgG-treated animals (Figure 6a–c). Activated microglia were quantified using magnified images (10–20 frames in each region per slice) (Figure 6a). There were a significantly lower number of activated microglia in Ab-T1-treated animals as compared to IgG-treated animals in both the hippocampal region and total brain area (Figure 6a–c). Furthermore, Figure 6d shows that there was no significant difference in the number of total microglia (activated and non-activated) in Ab-T1-treated animals as compared to control IgG-treated animals in the hippocampal region. We also examined the proteomic profile of cytokines in the brains of hTau.K257T/P301S mice treated with Ab-T1 compared to control IgG-treated mice using a protein array glass chip with fluorescence detection assay (RayBiotech, U.S.A). Cytokines and chemokines play important roles in inflammation, innate immunity, apoptosis, angiogenesis, cell growth, and differentiation. Ab-T1 treatment in hTau.K257T/P301S murine brains showed lower protein levels of a panel of inflammatory cytokines compared to control IgG treatment in most cytokines (Figure 6e).

### 5.7. Cortical Levels of Ab-T1-Treated hTau Tg Mice

**Figure 7:**
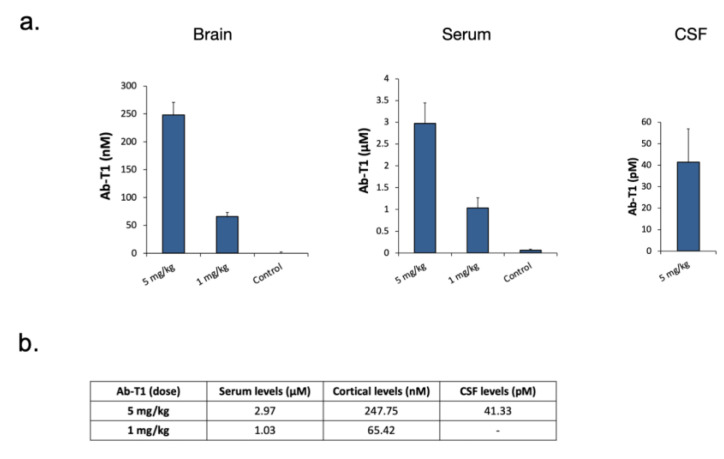
Ab-T1 levels in tauopathy Alzheimer mice. (**a**) Ab-T1 levels in serum, CSF, and brain of treated mice. Error bars represent standard deviation of the mean. (**b**) Summarize table of antibody levels in tauopathy Alzheimer mice treated with 5 or 1 mg/kg of Ab-T1.

Ab-T1 levels in the CSF, brain, and serum of mice were measured using a human IgG4 Elisa detection kit (Figure 7a). Figure 7b summarizes in the table the detectable levels of Ab-T1 in serum, cortex, and CSF of mice treated with two different doses of Ab-T1 (1 or 5 mg/kg).

## 6. Discussion

In the current study, we sought to study the effect of TREM2 agonism on Tau pathology and corroborate the in vivo experimental data with in vitro and ex vivo experiments designed to study the potential underlying mechanisms.

Ab-T1 was developed to target the ECD of human TREM2 [18], yet to exhibit a meaningful affinity to murine TREM2 so as to allow potential extrapolation of the data from the experimental studies to humans. We have previously shown in a set of studies in 5XFAD mice exhibiting a pure beta-amyloid pathology, that treatment with AbT-1 was associated with reduced amyloid burden including a change in plaque morphology as well as plaque-associated microglial activation and coverage [18]. However, the complex and mixed nature of the plaques in the AD brain, as well as the relevance of Tau as a driver of neurodegeneration, necessitates testing our antibody in the context of Tau-associated pathology. This is particularly important considering the inconsistent data that have been reported with regard to TREM2 ablation in different Tau-derived models and the lack of data with regard to the effects of microglial TREM2-mediated activation on Tau pathology and consequent neurodegeneration.

In tauopathies, Tau undergoes multiple post-translational modifications starting from hyperphosphorylation and leading to its misfolding and subsequent fibrilization and aggregation. There is ample data to support the role of extracellular misfolded Tau in mediating apoptotic and necrotic neuronal death [19,21,22,23]. We have thus aimed to test whether the potentiation of TREM2 expressing microglia would influence Tau uptake/clearance. As shown, a potentially beneficial effect of Ab-T1 could be related to the clearance of misfolded Tau as its uptake by microglia was significantly enhanced in the presence of the TREM2 antibody. This finding is also in line with the studies showing that TREM2 engagement was associated with increased uptake of oligomeric beta-amyloid and reduction in amyloid burden in vivo [16,17,18].

Additional mechanistic aspects that pathogenetically relate Tau to neurodegeneration involve its fibrillation, aggregation, seeding, and spreading [21]. We carried out a series of tests to serially study the effects of Ab-T1 on each of these aspects. We have shown in vitro that conditioning microglia with Ab-T1 was associated with attenuated seeding and aggregation of Tau in primary neurons from hTau mice. Although we cannot pinpoint a single mediating protecting factor, it appears that a collective panel of cytokines/chemokines inducible upon microglial TREM2 engagement with Ab-T1 inhibit spontaneous Tau seeding and aggregation in hTau mutated neurons. To further address the impact of Ab-T1 on endogenous Tau corruption and subsequent phosphorylation we performed an ex vivo organoid study using murine hTau brain slices grown in culture. Ab-T1 incubation/treatment of the cultures was associated with a significant reduction of Tau pathology evident by reduced p-Tau burden when brain extract-derived Tau was applied. This observation corroborates the previous studies that suggest that protection afforded by Ab-T1 by engulfing extracellular misfolded Tau and reducing seeding and aggregation contribute to the reduction in Tau pathology in this ex vivo organoid system.

We next aimed to test the effects of treatment in vivo with Ab-T1 on Tau spreading and propagation that could result from the protective mechanisms displayed previously. For this purpose, we exploited the model system where ipsilateral delivery of misfolded Tau drives propagation of misfolded Tau to the contralateral hemisphere in hTau transgenic mice. As shown in Figure 3, Ab-T1 treatment was associated with a significant reduction in NFT on the ipsilateral and contralateral sides. This protective effect could mirror the effects of the antibody on the clearance of extracellular misfolded Tau as well as on the paracrine effects imposed by the primed microglia.

To complete the totality of data with respect to the influence of Ab-T1 on a pure Tauopathy model we tested its effects when given therapeutically in vivo on cognition, Tau burden, and neurodegeneration in the double transgenic hTau murine model.

Treatment with Ab-T1 was associated with significant preservation of cognitive functions using the MWM. Importantly, these effects on cognition were associated with significant protection from neurodegeneration evident by improved synaptic preservation and reduced apoptotic and necrotic neuronal loss that was accompanied by the reduction in NFT, mirroring the reduced Tau burden. Interestingly, when assaying the number of activated hippocampal microglia number, we observed a significant reduction. In our previous study [18] testing Ab-T1 we observed an increase in the number of activated plaque-associated but not remote microglia attesting to the effect of this agonistic antibody on the plaque environment. In this tauopathy model, unlike the beta-amyloid-based models that exhibit extracellular plaques, the reduction in microglial number probably reflects the reduced Tau burden that is associated with reduced neuroinflammation. Accordingly, a proteomic analysis of hippocampal regions of Ab-T1-treated mice discloses a clear attenuation of the global neuroinflammatory program reflective of the reduction in activated microglia despite the employment of a TREM2 agonistic/activating antibody. Importantly, we measured Ab-T1 serum, CSF, and cortical levels, to understand if the brain levels achieved are reflective of the protective effects that the antibody has shown on microglial priming in vitro. The therapeutic doses employed in the in vivo studies achieved cortical levels that were at least as high as the concentrations needed to achieve microglial-mediated neuronal protection in vitro.

The effects of TREM2 expression in microglia in the context of Tau pathology have been inconsistent across the studies reported recently. The lack of a consistent effect is related to the different models and ages of the animals. In all studies, TREM2 deletion has been studied and thus the results mirror absent or aberrant versus constitutive expression of the membranal receptor. However, agonism of TREM2 in general and using an activating antibody specifically has not yet been tested and would clearly not be intuitively derived from the currently published data. We thus show here for the first time, that using our previously produced TREM2 activating antibody, Ab-T1, results in attenuation of Tau phosphorylation, propagation, and consequent neuroinflammation and neurodegeneration. Given the previously published data of Ab-T1 on the amyloid burden and its mechanism of action, the antibody could be an attractive mode of targeting both amyloid and tau pathologies in AD.

## Data Availability

The data of the study cohort may be requested from the corresponding author.
